# Predictive Accuracy of Magnetocardiography for Diagnosing Myocardial Ischemia in NSTE-ACS Patients With Residual Post-PCI Angina

**DOI:** 10.1016/j.jacadv.2025.102073

**Published:** 2025-08-19

**Authors:** Linqi Liu, Yilin Pan, Hai Gao, Yanyan Jin, Lanxin Feng, Zhao Ma, Huan Zhang, Shuwen Yang, Tingting Wu, Min Zhang, Mingduo Zhang, Feng Xu, Hongjia Zhang, Xiantao Song, Chenchen Tu

**Affiliations:** a Department of Cardiology, Beijing Anzhen Hospital, Capital Medical University, Beijing, China; b Arrhythmia Center, Fuwai Hospital, State Key Laboratory of Cardiovascular Disease, Chinese Academy of Medical Sciences & Peking Union Medical College/National Center for Cardiovascular Diseases, Beijing, China; c Department of Cardiac Surgery, Beijing Anzhen Hospital, Capital Medical University, Beijing, China; d Department of Cardiovascular Surgery, Fuwai Hospital, State Key Laboratory of Cardiovascular Disease, Chinese Academy of Medical Sciences & Peking Union Medical College/National Center for Cardiovascular Diseases, Beijing, China; e Department of Cardiology, Beijing Tongren Hospital, Capital Medical University, Beijing, China

**Keywords:** angina pectoris, optical pumped magnetometer magnetocardiography, least absolute shrinkage and selection operator, non-ST-segment elevation acute coronary syndrome, percutaneous coronary intervention

## Abstract

**Background:**

Post-percutaneous coronary intervention (PCI) angina affects 20% to 40% of non-ST-segment elevation acute coronary syndrome (NSTE-ACS) survivors but remains a clinical challenge due to its unpredictable course.

**Objectives:**

This study aimed to validate a rapid 90-second optically pumped magnetometer magnetocardiography (OPM-MCG) test for diagnosing post-PCI angina and develop a predictive model using dynamic MCG parameters.

**Methods:**

NSTE-ACS patients who underwent pre- and post-PCI OPM-MCG scans were included. Angina symptoms were assessed using the Seattle Angina Questionnaire Short Form-7, with post-PCI angina defined as a Seattle Angina Questionnaire angina frequency score <100 at 3 months. Changes in 65 MCG-derived parameters (Δ parameters) were evaluated. Predictors of post-PCI angina were identified via stratified bootstrap least absolute shrinkage and selection operator regression (1,000 iterations; variables retained if selected >70% of iterations). A final model was optimized and validated internally. Performance was evaluated using receiver operating characteristic curves, calibration, and decision curve analysis.

**Results:**

This study included 363 NSTE-ACS patients who underwent PCI, of whom 134 (36.9%) experienced post-PCI angina. Patients with post-PCI angina had higher body mass index, more frequent history of PCI, and greater use of oral nitrates. The OPM-MCG model, incorporating five Δ parameters (ΔRoART, ΔNPAmax, ΔMAmax, ΔCPPPPmax, and ΔCAmax), demonstrated strong diagnostic performance with an average area under curve of 0.85 (95% CI: 0.78-0.93) during bootstrapping. The final model achieved an area under curve of 0.79 (95% CI: 0.78-0.80) with 10-fold cross-validation.

**Conclusions:**

The OPM-MCG model provides a reliable, noninvasive, and quick tool for identifying post-PCI angina in NSTE-ACS patients, highlighting its clinical value for risk stratification, guiding personalized pharmacologic therapy, and follow-up strategies.

Globally, acute coronary syndrome (ACS) affect over 7 million individuals annually, with non-ST-segment elevation ACS (NSTE-ACS)—encompassing non-ST-segment elevation myocardial infarction and unstable angina—accounting for nearly 70% of cases.^1^, ^2^, ^3^ Despite advances in revascularization and pharmacotherapy, NSTE-ACS remains a major contributor to cardiovascular morbidity, with 2-year mortality rates ranging from 3.8% to 11.7% across regions.^4^ A critical unresolved challenge is the high prevalence of post-percutaneous coronary intervention (PCI) angina, affecting 20% to 40% of ACS survivors,^5^^,^^6^ which is associated with reduced quality of life, increased health care costs (up to 1.8-fold higher in the first year post-PCI),^7^ and heightened risks of psychological distress and adverse cardiovascular outcomes.^5^^,^^6^

Current assessment of post-PCI angina relies heavily on patient-reported tools such as the Seattle Angina Questionnaire (SAQ), a validated and widely used instrument that quantifies angina frequency, physical limitations, and disease perception.^8^ The SAQ has demonstrated strong reliability and validity and is correlated with long-term survival and hospitalization rates in patients with NSTE-ACS.^9^^,^^10^ While the SAQ correlates with long-term prognosis and hospitalization rates, its subjective nature limits its utility for objective diagnosis and mechanistic insight into ischemia. This underscores the need for noninvasive, reproducible biomarkers to complement patient-reported outcomes.

Here, we focus on optical pumped magnetometer-based magnetocardiography (OPM-MCG), a radiation-free technique that maps the heart’s magnetic activity, has emerged as a promising tool for detecting subclinical ischemia and stratifying risk in coronary artery disease.^11^, ^12^, ^13^, ^14^, ^15^, ^16^ Unlike traditional modalities (eg, stress echocardiography or nuclear imaging), MCG requires no physical exertion or contrast agents, enabling rapid (90 seconds) and repeatable assessments even in frail patients.^15^^,^^17^ Prior studies demonstrate MCG’s ability to identify ischemia in non-ST-segment elevation myocardial infarction and predict major adverse cardiovascular events,^18^, ^19^, ^20^ yet its role in diagnosing post-PCI angina—a condition driven by microvascular dysfunction, stent-related issues, or residual ischemia—remains unexplored. OPM-MCG uses laser-polarized alkali vapor sensors, which enable wearable applications and higher signal-to-noise ratios.

OPM-MCG differs from conventional Superconducting Quantum Interference Device (SQUID)-based MCG systems by enabling sensor placement in direct proximity to the chest surface, thereby improving spatial resolution to approximately 5 to 10 mm compared to the 10 to 20 mm typical of SQUID systems. Although OPM sensors exhibit slightly higher intrinsic noise levels (∼10-15 fT/Hz1/2) relative to SQUIDs (∼3-10 fT/Hz1/2), the substantially reduced source-to-sensor distance mitigates this difference and enhances cardiac signal capture. OPM-MCG has been applied in our previous studies.^21^^,^^22^

This study investigates the diagnostic accuracy of optical pumped magnetometer-based MCG (OPM-MCG) for identifying post-PCI angina in NSTE-ACS patients. By analyzing dynamic MCG parameter changes before and after PCI, we aim to develop an objective predictive model to guide personalized management and address a critical gap in postrevascularization care (Central Illustration).

**Central Illustration:**
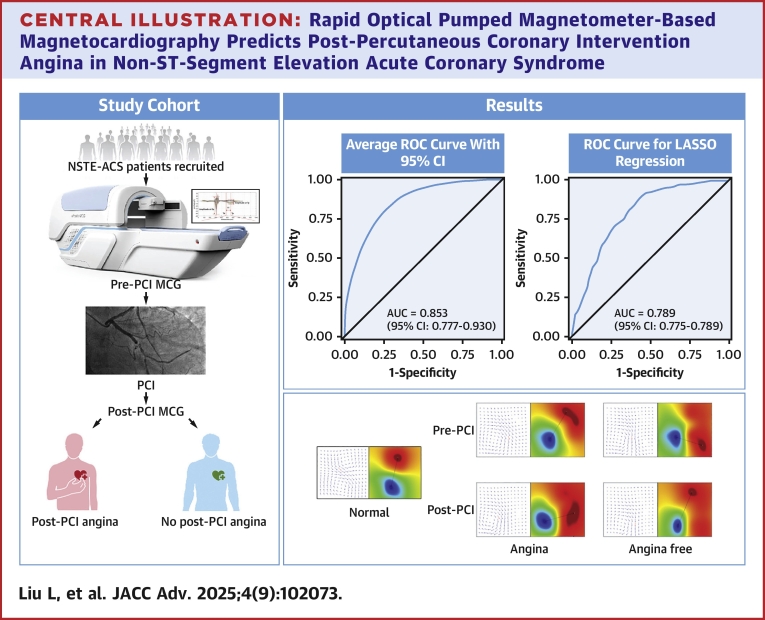
**Rapid Optical Pumped Magnetometer-Based Magnetocardiography Predicts Post-Percutaneous Coronary Intervention Angina in Non-ST-Segment Elevation Acute Coronary Syndrome** LASSO = least absolute shrinkage and selection operator; MCG = magnetocardiography; NSTE-ACS = non-ST-segment elevation acute coronary syndrome; ROC = receiver operating characteristic.

## Methods

### Design and study population

**Figure 1:**
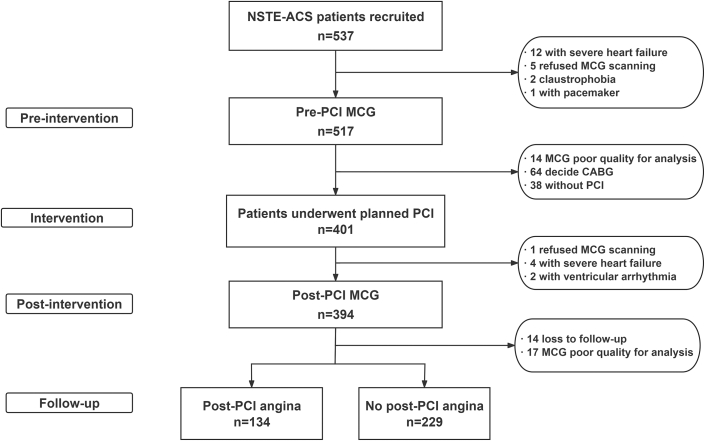
**The Flowchart** CABG = coronary artery bypass grafting; MCG = magnetocardiography; NSTE-ACS = non-ST-segment elevation acute coronary syndrome; PCI = percutaneous coronary intervention.

This prospective observational cohort study was conducted at Beijing Anzhen Hospital, Capital Medical University, from December 2022 to August 2023. Eligible participants were adults aged 18 to 80 years with a diagnosis of non-ST-segment elevation acute coronary syndrome (NSTE-ACS) and planned for invasive management. Exclusion criteria included: 1) patients with severe heart failure (LVEF <30% or NYHA functional class IV); 2) claustrophobia precluding MCG scanning; 3) ventricular arrhythmia (eg, sustained ventricular tachycardia/ventricular fibrillation); 4) conduction abnormalities (eg, bundle branch block and paced rhythm); 5) metallic implants or devices (eg, pacemakers); 6) poor-quality pre- or post-PCI MCG scans (automatically flagged by software); and 7) loss to follow-up or refusal to participate. Of 537 initially screened patients, 363 met inclusion criteria after exclusions (Figure 1).

All patients completed questionnaires on anginal symptoms (Seattle Angina Questionnaire Short Form [SAQ-7]) at baseline and were contacted to repeat this assessment 3 months after intervention. The questionnaires were administered by telephone by follow-up officers blinded to the MCG and baseline data. The cardiologists were not aware of the MCG results. All participants signed an informed consent agreeing to take MCG scanning. This study was approved by the Ethics Committee of Beijing Anzhen Hospital, Capital Medical University, and registered with the China Clinical Trial Registry (ChiCTR2300070211).

### OPM-MCG acquisition and analysis

A 36-channel OPM-MCG device (Miracle MCG, X-MAGTECH Medical) recorded cardiac magnetic activity using spin exchange relaxation-free sensors. To achieve an adequate signal-to-noise ratio and ensure the reliable extraction of cardiac magnetic waveforms through ensemble averaging, a recording duration of 90 seconds was selected. This duration captures approximately 100 cardiac cycles at resting heart rates, providing a robust dataset while maintaining patient comfort and minimizing the risk of motion artifacts. The 90-second protocol aligns with established standards in clinical and research applications of MCG. All MCG scans were performed under resting conditions without sedation, and patients were not instructed to withhold medications prior to scanning.

Sensors were arranged in a 6 × 6 grid over the chest, with residual direct current fields <1.5 nT. The sensitivity of the OPM sensor is below 15 fT/Hz1/2 (Supplemental Figure 1), with a dynamic range of ±3 nT. The bandwidth used by the OPM-MCG device is 1 to 40 Hz, with a sampling rate of 200 Hz. The direct current residual magnetic field in the MCG measurement area is maintained below 1.5 nT.

The signal quality of the MCG device is automatically assessed through dedicated MCG software, ensuring accurate and reliable measurements. These assessments encompass evaluating signal purity via signal-to-noise ratio calculation, determining noise impact by analyzing frequency domain energy distribution, and identifying aberrant signals or noise through the computation of statistical distribution characteristics. Furthermore, as depicted in Supplemental Figure 1A, operators can directly observe signal consistency across all 36 channels during the signal acquisition process. In instances of significant interference, the operator retains the authority to reject the examination results. Since MCG scans were performed while participants remained at rest, stable and consistent data can be collected. Gradiometer noise suppression, empirical mode decomposition, automated filtering (50 Hz powerline notch and band-pass), and independent component analysis were applied to the data for artifact removal. Following these preprocessing steps, R-peak-centered data segments were extracted and averaged to improve analytical accuracy. The resulting data were further processed into three distinct components for comprehensive evaluation: a butterfly diagram, which visually represents the temporal changes in the magnetic field; a magnetic field map, which illustrates the spatial distribution of the magnetic fields generated by the heart; and a pseudo current density map, which provides insights into the underlying electrical activity of the myocardium.^23^

**Figure 2:**
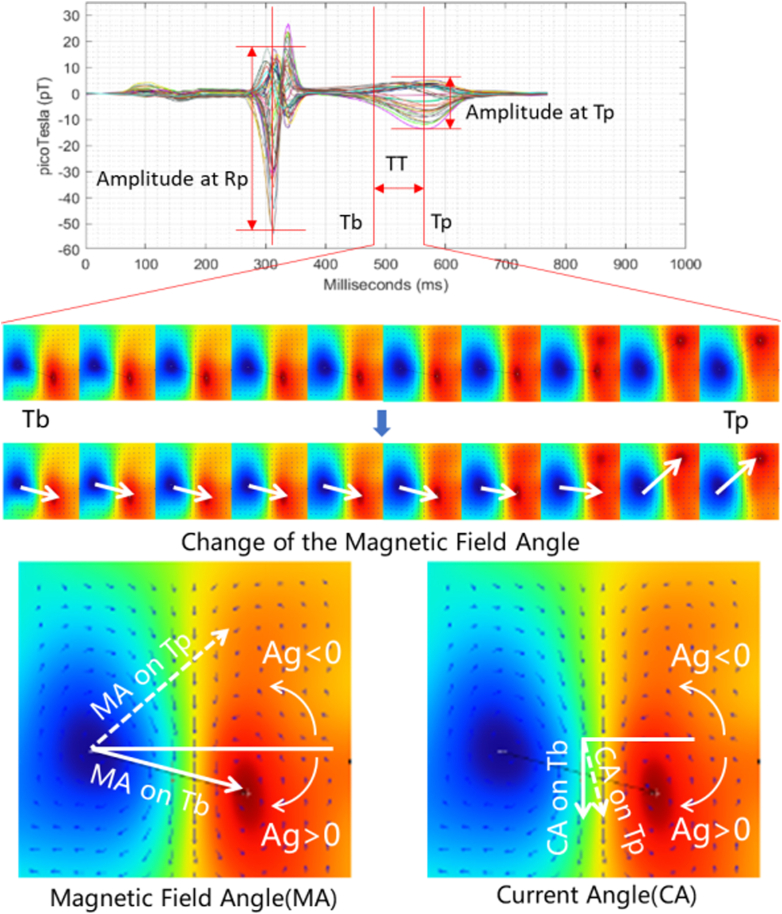
**Visualization of Fundamental Parameter Definitions** Illustration of key fundamental parameters extracted from OPM-MCG signals, using a representative patient's MCG results as an example. Ag = angle; CA = current angle; MA = magnetic field angle; Rp = R-peak; Tb = T-begin; Tp = T-peak; TT = TT segment.

Upon completion of MCG data acquisition, the software performs postprocessing to extract a total of 65 parameters. Using the example of a representative patient's MCG results, Figure 2 illustrates the definitions of key fundamental parameters, such as magnetic field angle, current angle, and amplitude. It is noteworthy that all 65 parameters are derived from these basic parameters through calculations based on the explanations provided in Supplemental Table 1.

### Invasive Coronary Angiography and PCI process

Invasive coronary angiography was conducted on all major coronary arteries using the femoral or radial artery approaches with Philips Allura and Azurion catheter equipment, and the intervention was performed according to the 2023 European Society of Cardiology Guidelines for the management of acute coronary syndromes.^3^ Multivessel disease patients underwent staged interventions, with post-PCI MCG performed after final revascularization. Quantitative Coronary Angiography (QCA, Medis QAngio, Medis) was utilized for quantitative analysis of coronary artery stenosis.

### Outcome definitions

Post-PCI angina was defined as a Seattle Angina Questionnaire-Angina Frequency (SAQ-AF) score <100 at the 3-month follow-up. The SAQ-AF domain quantifies the frequency and burden of angina symptoms. This subscale assesses the frequency of angina episodes over the preceding 4 weeks, with scores ranging from 0 (daily angina) to 100 (no angina). A score <100 indicates at least 1 episode of angina during the follow-up period. Missing physical limitation scores were imputed using the subject’s mean value from the nonmissing items within the same exertional level.^24^

Major adverse cardiac or cerebrovascular event (MACCE) was a composite endpoint occurring within 1 year post-PCI, including death from cardiovascular causes, myocardial infarction, stroke, revascularization procedure, and hospital admission for heart failure. Follow-up assessments were standardized to occur at 90 ± 7 days and 1 year ±7 days post-PCI to minimize recall bias. Patients lost to follow-up (n = 14) were excluded from the primary analysis.

### Statistical analysis

Data distribution was first assessed using the Shapiro-Wilk test and visualized through quantile–quantile (QQ) plots. Continuous variables were expressed as either mean ± SD if normally distributed or median (IQR) if non-normally distributed. The normality of data was tested using the Shapiro-Wilk test, and group comparisons were made using the appropriate statistical tests. For normally distributed continuous variables, the Student’s *t*-test was employed, while non-normally distributed variables were compared using the Mann-Whitney U test. Categorical variables were analyzed using the chi-square test or Fisher exact test. Paired samples were compared using the paired t-test or Wilcoxon signed-rank test, as appropriate. CIs were not adjusted for multiple comparisons and should be interpreted with caution. All statistical tests were two-tailed.

The OPM-MCG data were processed to generate magnetic field and current density maps, yielding 65 parameters (Supplemental Table 1) that quantified current dipole stability in the T-wave segment (T onset to T peak), consistent with previous studies by Park et al^25^ and Pena et al.^26^ These parameters were used to evaluate post-PCI angina and MACCE prediction by calculating 65 preoperative to postoperative dynamic change indicators. Data preprocessing removed near-zero variance variables (variance threshold = 0.10) and highly correlated predictors (Pearson correlation >0.9) to minimize noise and multicollinearity. A hybrid-stratified bootstrap-least absolute shrinkage and selection operator (LASSO) approach (1,000 iterations) was used to identify predictors: stratified resampling preserved outcome proportions, while LASSO with 10-fold cross-validation selected nonzero coefficients. Variables selected ≥700 times were retained. The final LASSO model used repeated cross-validation (10-fold × 10 repeats) to optimize λ (range: 0.0001-10) via area under curve (AUC)-receiver operating characteristic (ROC) curve, with predictors standardized premodeling. Internal validation assessed discrimination (cross-validated AUC with DeLong’s 95% CI and bootstrap ROC curves), calibration, and clinical utility (decision curve analysis net benefit). Visualizations included variable importance plots (top 10 predictors) and ROC curves annotated with formatted AUC values.

All statistical analyses were conducted using R software (version 4.2.2).

## Results

### Baseline characteristics

The study enrolled 363 patients with NSTE-ACS (76.9% male, mean age 57.8 ± 11.0 years). All participants underwent pre- and post-PCI MCG scans, with post-PCI assessments completed within 14 days of intervention. Of the 363 participants, 134 (36.9%) experienced post-PCI angina, as determined by a follow-up SAQ-AF score <100. Baseline characteristics are summarized in Table 1. Patients with post-PCI angina had a higher body mass index (BMI) and a greater history of previous PCI procedures. Additionally, these patients had a significantly higher rate of oral nitrate use compared to those without post-PCI angina.

**Table 1:** Baseline

Variables	Total (N = 363)	Post-PCI Angina (n = 134, 36.9%)	No Post-PCI Angina (n = 229, 63.1%)	*P* Value
Age, y	57.8 ± 11.0	59.2 ± 10.5	57.0 ± 11.2	0.063
Male	279 (76.9)	100 (74.6)	197 (78.2)	0.440
BMI, kg/m^2^	26.47 ± 3.54	26.92 ± 3.91	26.18 ± 3.28	0.036
Hypertension	216 (59.5)	82 (61.2)	134 (58.5)	0.616
Dyslipidemia	182 (50.1)	65 (48.5)	87 (51.1)	0.635
Diabetes mellitus	142 (39.1)	82 (44.8)	60 (35.8)	0.091
Heart failure	44 (12.1)	16 (12.0)	28 (12.2)	0.936
Atrial fibrillation	7 (1.9)	2 (1.5)	5 (2.2)	0.644
Grace score	110.28 ± 27.54	112.25 ± 27.88	109.13 ± 27.34	0.298
Grace score class				
Low risk class	221 (60.9)	80 (59.7)	141 (61.6)	0.939
Moderate risk class	87 (24.0)	33 (24.6)	54 (23.6)	
High risk class	55 (15.1)	21 (15.6)	34 (14.8)	
Current smoker	140 (38.6)	54 (40.3)	86 (37.6)	0.604
Family history of CAD	178 (49.0)	70 (52.2)	108 (47.2)	0.350
eGFR, mL/min	91.84 ± 29.70	90.20 ± 31.44	92.80 ± 28.66	0.423
LVEF, %	61.57 ± 7.23	61.97 ± 7.38	61.34 ± 7.15	0.424
Previous MI	54 (14.9)	22 (16.4)	32 (14.0)	0.528
Previous PCI	71 (19.6)	34 (25.4)	37 (16.2)	0.033
NSTEMI	62 (17.1)	23 (17.2)	39 (17.0)	0.974
CKMB, ng/mL	1.4 (0.9-2.2)	1.35 (0.9-2)	1.4 (0.9-2.3)	0.213
Tn I, pg/mL	5.7 (3.1-18.1)	5.3 (2.8-14.9)	5.8 (3.2-20.0)	0.572
BNP, pg/mL	47 (20-109)	51.2 (25-98.4)	45 (19-115)	0.371
Target vessel				
RCA	153 (42.2)	63 (47.0)	90 (39.3)	0.151
LAD	182 (50.1)	60 (44.8)	122 (53.3)	0.118
LCX	80 (22.0)	27 (20.2)	53 (23.1)	0.507
No. of diseased vessels				
1-vessel disease	118 (32.5)	41 (30.6)	77 (33.6)	0.575
2-vessel disease	114 (31.4)	40 (29.9)	74 (32.3)	
3-vessel disease	131 (36.1)	53 (39.6)	78 (34.1)	
Residual stenosis	107 (29.5)	40 (29.9)	67 (29.3)	0.90
Medication				
Aspirin	363 (96.1)	134 (97.0)	229 (95.6)	0.509
Any statin	355 (97.8)	132 (98.5)	223 (97.4)	0.480
P2Y12 receptor inhibitor	254 (70.0)	87 (64.9)	176 (72.9)	0.109
Beta-blocker	363 (53.7)	134 (50.8)	229 (55.5)	0.385
ACE inhibitor	278 (76.6)	178 (80.6)	170 (74.2)	0.167
ARB	118 (32.5)	42 (31.3)	76 (33.2)	0.717
Calcium channel blocker	101 (27.8)	40 (30.0)	61 (26.6)	0.510
Oral nitrates	95 (26.2)	45 (33.6)	50 (21.8)	0.014
Planned PCI times	1 (1-1)	1 (1-1)	1 (1-1)	0.565
Pre-PCI MCG to PCI interval, d	2 (1-6)	2 (1-5)	2 (1-6)	0.745
Pre-PCI MCG to PCI interval, d	2 (1-7)	2 (1-7)	2 (1-8)	0.890

Values are mean ± SD or n (%).

ACE = angiotensin-converting enzyme; ARB = angiotensin receptor blocker; BNP = brain natriuretic peptide; BMI = body mass index; CAD = coronary artery disease; CKMB = creatine kinase-myocardial band; eGFR = estimated glomerular filtration rate; LAD = left anterior descending artery; LCX = left circumflex artery; LVEF = left ventricular ejection fraction; MCG = magnetocardiography; MI = myocardial infarction; NSTEMI = non-ST-segment elevation myocardial infarction; PCI = percutaneous coronary intervention; RCA = right coronary artery; Tn I = troponin I.

Baseline and follow-up SAQ-7 scores are presented in Table 2 and Supplemental Table 2. Physical limitation scores were missing for 23 patients at baseline and 33 patients during follow-up due to pre-existing physical limitations that precluded self-assessment. Patients with post-PCI angina exhibited lower baseline SAQ scores compared to those without angina.

**Table 2:** Baseline, Follow-Up, and Change in SAQ-7 Scores

	Total	Post-PCI Angina	No Post-PCI Angina	*P* Value
Baseline SAQ-7				
Physical Limitation score, mean ± SD	57.17 ± 21.60	53.50 ± 23.30	59.30 ± 20.30	0.0171
Angina Frequency score	53.16 ± 17.47	47.99 ± 14.56	56.18 ± 18.33	<0.001
Quality of Life score	48.69 ± 18.89	43.99 ± 17.45	51.45 ± 19.20	0.0003
Summary Score	52.23 ± 12.26	46.78 ± 12.41	55.42 ± 11.00	<0.001
Follow-up SAQ				
Physical Limitation score	80.23 ± 14.58	71.36 ± 15.02	85.32 ± 11.60	<0.001
Angina Frequency score	88.45 ± 17.0	68.71 ± 12.83	100.00 ± 0.00	<0.001
Quality of Life score	77.08 ± 15.03	68.74 ± 14.85	81.96 ± 12.86	<0.001
Summary Score	82.19 ± 12.26	69.91 ± 9.24	89.38 ± 6.94	<0.001
Change in SAQ Scores				
Change in Physical Limitation score	23.07 ± 19.04	17.83 ± 21.66	26.07 ± 16.68	<0.001
Change in Angina Frequency score	35.29 ± 21.32	20.73 ± 17.97	43.82 ± 18.33	<0.001
Change in Quality of Life score	28.38 ± 15.98	24.75 ± 18.11	30.51 ± 14.21	<0.001
Change in Summary Score	29.18 ± 12.57	21.45 ± 11.95	33.71 ± 10.57	<0.001

Values are mean ± SD.

PCI = percutaneous coronary intervention; SAQ = Seattle Angina Questionnaire.

### Feature selection and model development

**Figure 3:**
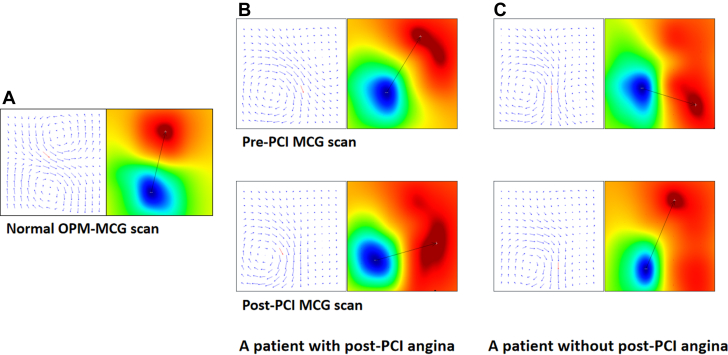
**Diagram of Optical Pumped Magnetometer-Based Magnetocardiography Scan in T-Peak** (A) MFM and pseudo CDM at T peak obtained from OPM-MCG scan of a healthy subject. (B) MFM and pseudo CDM at T peak obtained from OPM-MCG scan of a patient with post-PCI angina. (C) MFM and pseudo CDM at T peak obtained from OPM-MCG scan of a patient without post-PCI angina. CDM = current density map; OPM-MCG = optical pumped magnetometer-based magnetocardiography; MCG = magnetocardiography; MFM = magnetic field map; PCI = percutaneous coronary intervention.

Representative OPM-MCG scan changes are illustrated in Figure 3 and Supplemental Videos. Figure 3 illustrates the magnetic field map and current density map at the T-peak for a healthy subject, as well as for a patient with angina and a patient without angina, both before and after PCI. The supplementary materials contain three videos corresponding to the OPM-MCG scans of these 3 representative subjects. As observed in Supplemental Video 1, the magnetic field angle of the TT segment in the MCG of a healthy subject remains stable around −45°, with no significant changes in the shape or area of the positive and negative poles. Supplemental Video 2 presents the MCG scan of a patient who experienced angina post-PCI. It can be observed that the magnetic field angles of the TT segment in the MCG demonstrate a counterclockwise rotation both before and after the PCI. The shapes and areas of the positive and negative poles exhibit minimal differences. By the peak of the T wave, the magnetic field angle has returned to the normal range (−45° to −86°) pre-PCI, whereas post-PCI, the magnetic field angle remains outside the normal range. Supplemental Video 3 shows MCG scans of a patient who did not experience angina post-PCI. It can be seen that pre-PCI, the magnetic field angles in the TT segment of the MCG exhibit a clockwise rotation, accompanied by the emergence of a multipolar pattern in the positive pole. The negative pole displays an irregular shape with a noticeable increase in area, and at the peak of the T wave, the magnetic field angle deviates, falling outside the normal range. Post-PCI, the TT segment of the MCG demonstrates stable magnetic field angles, all within the normal range, while the shape of the negative pole is regular with no significant changes observed.

**Figure 4:**
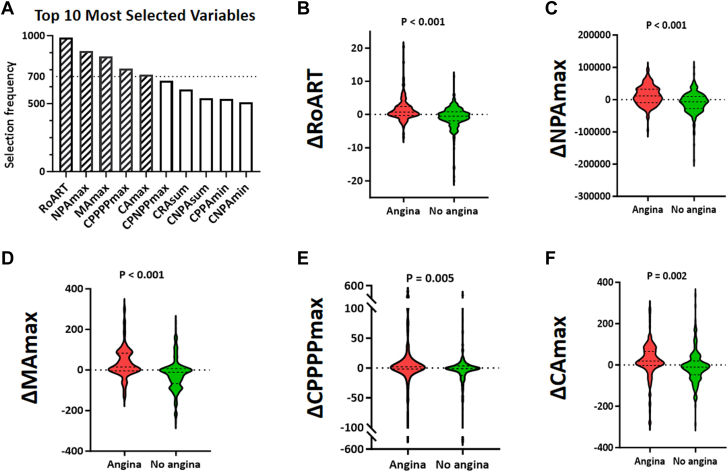
**Selection Frequency and Longitudinal Changes of Top Parameters** (A) The variables with the highest selection frequencies. (B) RoART values between patients with and without angina. (C) NPAmax values between patients with and without angina. (D) MAmax values between patients with and without angina. (E) CPPPPmax values between patients with and without angina. (F) CAmax values between patients with and without angina. PCI = percutaneous coronary intervention.

A comprehensive evaluation of 65 Δ parameters was conducted to identify predictors associated with angina. Parameters related to magnetic field amplitudes (eg, ΔRoART) and negative pole area (eg, ΔNPAmax) showed the strongest associations (Supplemental Table 3). To ensure the robustness and stability of these predictors, a feature selection process was implemented using 1,000 bootstrap samples combined with 10-fold cross-validation LASSO regression. This procedure aimed to identify variables that were consistently selected across multiple resampled datasets. The top variables shown in Figure 4, represent the most important parameters identified. Through the bootstrapping procedure, five Δ parameters—RoART, NPAmax, MAmax, CPPPPmax, and CAmax (Table 3)—consistently emerged as predictors, each selected in over 70% of bootstrap iterations. Bootstrap resampling (1,000 iterations) confirmed predictor stability: ΔRoART and ΔNPAmax were reselected in 99% and 89% of samples, respectively, and all five variables appeared in ≥70% of samples; their median standardized β’s ranged from 0.002 to 0.127 with narrow 95% bootstrap CIs (Supplemental Table 4).

**Table 3:** Diagnostic Performance of the LASSO Model

MCG Parameters	Definitions
RoART	The ratio of magnetic field amplitudes at R-peak and T-peak
NPAmax	The maximum value of the negative pole area at intervals of a certain time τ within TT segment
MAmax	The maximum magnetic field angle at intervals of a certain time τ within TT segment
CPPPPmax	The maximum value of changes in the position of the positive pole point at intervals of a certain time τ within TT segment
CAmax	The maximum current angle at intervals of a certain time τ within TT segment

τ = one-tenth of the time interval between TT segment; δ = change value; A = angle/magnitude/area; bp = baseline to post-P wave; C = current; CN = negative pole; CP = positive pole; D = distance; LASSO = least absolute shrinkage and selection operator; M = magnetic field; max = maximum; MCG = magnetocardiography; min = minimum; P = position; sum = sum of all values; R = R peak; Ro = ratio; Rp = R peak; T = T peakt; Tp = T peak; TT = TT segmen.

**Figure 5:**
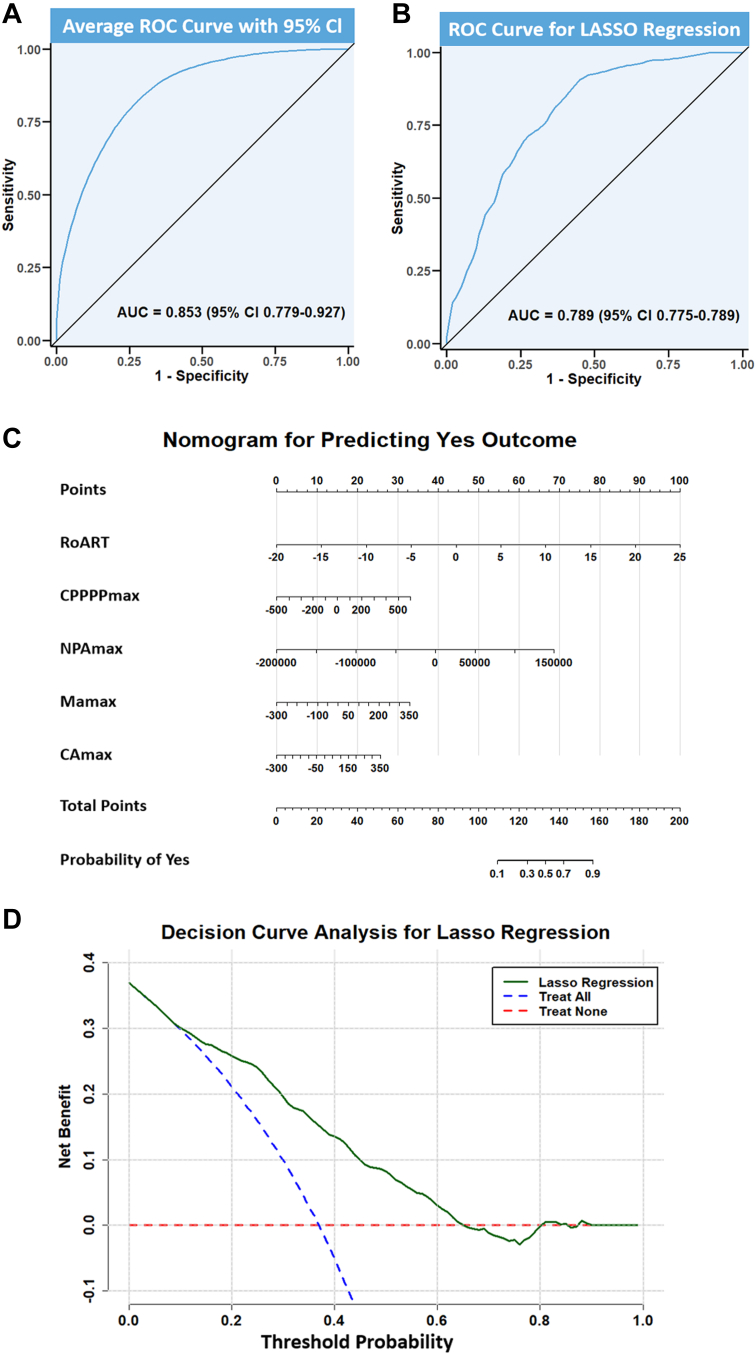
**Model Performance, Clinical Utility, and Prediction Tool for Post-Percutaneous Coronary Intervention Angina** (A) ROC curve evaluating average diagnostic performance across 1,000 bootstrap iterations. (B) ROC curve of the final LASSO regression model for predicting post-PCI angina. (C) Decision curve analysis (DCA) showing clinical net benefit across threshold probabilities for post-PCI angina prediction models. (D) Nomogram based on the final LASSO model to predict post-PCI angina. AUC = area under curve; LASSO = least absolute shrinkage and selection operator; MCG = magnetocardiography; PCI = percutaneous coronary intervention; ROC = receiver operating characteristic.

The diagnostic performance of the models, assessed using ROC curves and the AUC metric, showed strong discriminative ability with an average AUC of 0.85 (95% CI: 0.78-0.93) across bootstrap iterations (Figure 5A).

### Development of the diagnostic model

Using the five stable Δ parameters identified in the bootstrapping phase, a final LASSO regression model was developed. A 10-fold cross-validation procedure was used to determine the optimal regularization parameter (λ_min). The final model was fitted using λ_min, which ensures the optimal balance between model complexity and predictive accuracy. The selected variables remained consistent with those identified during the bootstrapping phase, confirming their significance in predicting angina. The final LASSO regression model achieved an AUC of 0.79 (95% CI: 0.78-0.80) (Figure 5B).

The calibration of the final model was assessed through calibration curves, which showed good agreement between predicted probabilities and observed outcomes. A nomogram based on the final model’s coefficients was developed as a practical tool for individualized risk prediction of angina (Supplemental Figure 2).

Additionally, using the same method, only using pre-PCI parameters (AUC = 0.67, 95% CI: 0.65-0.69) or post-PCI parameters (AUC = 0.73, 95% CI: 0.71-0.75) showed limited diagnostic performance (Supplemental Table 5, Supplemental Figure 3).

## Secondary outcomes

At 1-year follow-up, MACCE occurred in 37 patients (10.2%) overall, including 18 of 134 (13.4%) in the post-PCI angina group and 19 of 229 (8.3%) in the no-angina group (*P* = 0.119); given the low event count, we did not pursue multivariable modeling for this endpoint.

### Sensitivity analysis

Sensitivity analysis with elastic-net regularization (α tuned 0-1) on all 65 Δparameters produced an AUC of 0.77 (95% CI 0.76-0.79), calibration-in-the-large of −0.001, and calibration slope of 1.08 (Supplemental Figure 4, Supplemental Table 5). These metrics were comparable to the primary LASSO model (AUC 0.79; slope 0.92), indicating that model performance was robust to the choice of penalization technique.

Spline analysis confirmed that the relationships of ΔRoART, ΔNPAmax, ΔMAmax, ΔCPPPPmax, and ΔCAmax with post-PCI angina were essentially linear (all Pnon-lin ≥0.05; Supplemental Table 6). Smoothed curves are shown in Supplemental Figure 5.

Exploratory diagnostics confirmed ΔRoART and ΔNPAmax as the dominant contributors: they have the largest standardized β values. Permutation importance (Supplemental Figure 6B) showed that permuting ΔNPAmax alone reduced cross-validated AUC by 0.14, whereas permuting any of the other four predictors changed AUC by ≤0.001, indicating that ΔNPAmax provides the dominant discrimination signal while the remaining variables contribute primarily to calibration.

Calibration was good overall (intercept <0.001, slope = 1.03) and similar across sex (female 0.298/1.65; male −0.085/0.90) and BMI strata (<26 kg/m^2^ –0.29/1.27; ≥26 kg/m^2^ 0.26/0.86) (Supplemental Table 7).

## Discussion

This study establishes OPM-MCG, a rapid, noninvasive magnetocardiography platform, as a novel diagnostic tool for post-PCI angina in NSTE-ACS patients. The findings highlight the diagnostic accuracy of the OPM-MCG model and provide new insights into its clinical utility.

Our results revealed that 36.9% of patients experienced post-PCI angina, a prevalence consistent with prior reports on angina incidence after coronary revascularization.^5^^,^^6^ Patients with post-PCI angina exhibited higher BMI, a greater history of previous PCI, and increased use of oral nitrates, suggesting these factors may contribute to or correlate with postprocedural angina. The presence of post-PCI angina underscores the persistent burden of angina despite revascularization and highlights the importance of accurate diagnostic tools for early identification and management.

In previous study, MCG’s ability to detect microvascular dysfunction^27^ provides a plausible mechanistic explanation for its diagnostic utility in post-PCI angina. Microvascular dysfunction—characterized by impaired coronary flow reserve, endothelial dysfunction, or vasomotor abnormalities—is recognized as a driver of persistent angina after PCI, even in the absence of significant epicardial stenosis.^28^^,^^29^ While our OPM-MCG model likely captures subtle ischemia caused by microcirculatory disturbances, larger-scale studies are warranted to validate its diagnostic precision and pathophysiological relevance.

Collison et al’s study^30^ identified predictors of post-PCI angina, with their reported incidence (38.3%) and detrimental impact on quality of life aligning closely with our findings (36.9%). Their work emphasized procedural factors linked to intracoronary functional improvements, notably smaller post-PCI fractional flow reserve (FFR) gains (post-PCI FFR minus pre-PCI FFR) as a predictor of persistent angina, alongside the association between lower baseline SAQ. Notably, Collison et al utilized invasive intracoronary physiology to link hemodynamic changes to angina relief, whereas our OPM-MCG model provides a noninvasive alternative by quantifying dynamic magnetic field parameters. Their study observed that patients with post-PCI angina did not exhibit significant differences in post-PCI FFR or index of microcirculatory resistance. While Collison et al’s study highlights the importance of invasive intracoronary functional markers, our OPM-MCG technology offers a noninvasive and highly effective alternative to invasive procedures that significantly reduces patient harm and significantly shortens in-operating room testing time. Together, these studies advocate a multimodal integrated strategy that combines physiologic guidance during PCI with postoperative angina monitoring to provide a personalized treatment plan and ultimately an improved prognosis for this high-risk patient population.

Our findings also align with and extend the evolving body of MCG research. Park et al^25^ demonstrated that MCG outperformed electrocardiography, echocardiography, and troponin-I for detecting coronary artery disease in patients with acute chest pain, highlighting its diagnostic potential in early ischemia. More recent studies have expanded MCG’s role in identifying non-ST-elevation myocardial infarction,^12^^,^^13^ detecting exercise-induced ischemia,^14^ and predicting long-term outcomes in acute myocardial infarction patients.^18^^,^^19^ Across these works, features such as magnetic field angle shifts, current distribution, and repolarization heterogeneity were consistently associated with ischemic burden and prognosis—supporting our model's inclusion of angle and pole-based parameters. By focusing specifically on post-PCI angina, our study adds a new dimension to this literature, suggesting that OPM-MCG may help distinguish between macrovascular and microvascular ischemia and further validate its use in real-world clinical decision-making.

A previous study from our team^22^ demonstrated that OPM-MCG effectively evaluates myocardial ischemia in patients with borderline coronary lesions (50% to 70% stenosis), underscoring its utility for functional assessment of coronary stenosis. While the current study focuses on postrevascularization angina evaluation, the earlier work addressed ischemia detection in preinterventional scenarios, collectively highlighting MCG’s versatile clinical applicability across the disease continuum. Differences in study populations and endpoints—chronic coronary syndrome with borderline lesions vs post-PCI angina in ACS patients in the current study—likely account for the variation in selected MCG parameters. While previous study emphasized magnetic angle and pole area changes, both models highlight the relevance of angle variability and pole morphology in myocardial ischemia.

Another work from our team^21^ validated the utility of OPM-MCG in detecting myocardial perfusion deficits using single-photon emission computed tomography as the reference standard. In that work, LASSO regression identified five critical MCG parameters—RoART+, RTA, MAmax, CCAmax, and CPPPATbp—to train machine learning models (random forest, decision tree, and support vector machine). These models achieved strong diagnostic performance (AUC: 0.780-0.804) with high sensitivity (82.6% to 91.3%), though specificity remained modest (10.0-50.0%). Notably, several features from the single-photon emission computed tomography-based model overlap conceptually with those in our current study. For instance, RoART+, MAmax, and CPPPATbp correspond closely to RoART, MAmax, and CPPPPmax, respectively. This overlap reinforces the role of field amplitude ratios and angular/polar dynamics as robust, ischemia-sensitive features across different reference standards and patient populations.

Integrating findings across our three studies suggests that OPM-MCG may offer more than ischemia detection. In our current study, similar residual stenosis rates between patients with and without post-PCI angina point to microvascular dysfunction as a likely contributor to persistent symptoms. Parameters such as CPPPPmax and CAmax may serve as noninvasive markers of microvascular ischemic burden, reflecting spatial or directional dispersion of cardiac magnetic fields. In contrast, the parameters emphasized in previous study appear more reflective of fixed epicardial obstruction in chronic coronary syndrome patients.^22^ These comparisons highlight the potential of OPM-MCG to distinguish between macrovascular and microvascular ischemia. Future multicenter studies incorporating anatomical and functional validation are needed to refine these models and support their use in personalized post-PCI management.

Studies by Bang et al^18^ and Korhonen et al^20^ demonstrated MCG’s potential in predicting long-term outcomes for acute myocardial infarction patients. Bang et al linked non-dipole T-wave patterns to increased major adverse cardiovascular event risk, while Korhonen et al associated fragmented QRS with adverse events. Our study explored MCG parameters for MACCE prediction, but the variables showed nominal significance, insufficient for a robust model. Despite our larger cohort (363 ACS patients vs 124 and 158 in their studies), further recruitment is needed to identify reliable MCG biomarkers for MACCE. These findings highlight MCG’s promise in cardiovascular risk stratification, warranting further validation for clinical integration.

Building on these findings, future research should explore the role of OPM-MCG in predicting other post-PCI complications, such as recurrent ischemia or arrhythmias. Additionally, incorporating OPM-MCG parameters with clinical, laboratory, and imaging data may enhance predictive accuracy and broaden its clinical applications. Visual features observed in magnetic field maps—such as angle shifts and pole morphology—may also support automated ischemia detection. Applying artificial intelligence, including machine learning or deep learning algorithms, to MCG images could enable real-time identification of these patterns, improving diagnostic efficiency. Longitudinal studies are warranted to evaluate the impact of OPM-MCG-guided management on patient outcomes, quality of life, and health care utilization. In clinical practice, patients identified by OPM-MCG as high-risk for persistent angina could benefit from intensified antianginal regimens, early lifestyle modifications, or closer follow-up schedules to optimize symptom control and prevent adverse events.

### Study Limitations

This study has several limitations that must be acknowledged. Firstly, the restriction of the equipment in a single center may limit the generalizability of the findings to other populations or health care settings. Secondly, the relatively small sample size for patients with post-PCI angina may impact the precision of certain estimates. Thirdly, large-scale studies are needed to better assess the incidence of post-PCI angina and its association with long-term adverse cardiovascular outcomes. Due to the low MACCE event count, we did not perform multivariable modeling for this endpoint. Finally, it should be noted that the definition of positive and negative poles we currently use differs from the Rome Biomag Conference in 1981 standard, and there are currently multiple types of MCGs globally, and the current methodology for analyzing angina has not been compared head-to-head with other MCG devices.

## Conclusions

This study demonstrates the diagnostic accuracy of OPM-MCG for identifying post-PCI angina in patients with NSTE-ACS. The developed model, based on dynamic changes in MCG parameters, offers a reliable and clinically meaningful tool for detecting myocardial ischemia. These findings suggest that OPM-MCG may enhance risk stratification and provide valuable guidance for tailoring pharmacologic treatment and follow-up strategies in post-PCI patients, ultimately improving the management of this high-risk population.

Perspectives **COMPETENCY IN MEDICAL KNOWLEDGE:** Residual angina following PCI in patients with NSTE-ACS remains a major clinical challenge, often associated with microvascular dysfunction or incomplete revascularization. OPM-MCG offers a rapid, noninvasive, radiation-free diagnostic modality capable of detecting subtle myocardial ischemia, providing mechanistic insights beyond conventional clinical assessment tools.

**TRANSLATIONAL OUTLOOK:** Future multicenter, prospective studies are warranted to validate the use of OPM-MCG in broader populations and to evaluate its impact on long-term outcomes, health care utilization, and personalized post-PCI therapeutic strategies.

## Funding support and author disclosures

This work was supported by Coordinated Innovation of Scientific and Technological in the Beijing-Tianjin-Hebei Region (Z231100003923008), the Beijing Nova Program (20220484222), Capital’s Funds for Health Improvement and Research (2024-2-2066), the Beijing Hospitals Authority “Sailing” Program (YGLX202323), the Beijing Hospitals Authority’s Ascent Plan (DFL20220603), the high-level public health technical talent construction project of Beijing Municipal Health Commission (Leading Talent-02-01) and the Project of the Beijing Lab for Cardiovascular Precision Medicine (PXM2018_014226_000013). The authors have reported that they have no relationships relevant to the contents of this paper to disclose.

## References

[1] Bhatt D.L., Lopes R.D., Harrington R.A. (2022). Diagnosis and treatment of acute coronary syndromes: a review. JAMA.

[2] Gulati M., Gulati P.D., Levy D. (2021). 2021 AHA/ACC/ASE/CHEST/SAEM/SCCT/SCMR Guideline for the Evaluation and Diagnosis of Chest Pain: A Report of the American College of Cardiology/American Heart Association Joint Committee on Clinical Practice Guidelines. J Am Coll Cardiol.

[3] Rossello X., Dan G.-A., Dweck M.R. (2023). 2023 ESC guidelines for the management of acute coronary syndromes. Eur Heart J.

[4] Bueno H., Rossello X., Pocock S.J. (2019). In-hospital coronary revascularization rates and post-discharge mortality risk in non–ST-segment elevation Acute Coronary Syndrome. J Am Coll Cardiol.

[5] Plomondon M.E., Magid D.J., Masoudi F.A., J (2008). Association between angina and treatment satisfaction after myocardial infarction. J Gen Intern Med.

[6] Beinart S.C., Sales A.E., Spertus J.A., Plomondon M.E., Every N.R., Rumsfeld J.S. (2003). Impact of angina burden and other factors on treatment satisfaction after acute coronary syndromes. Am Heart J.

[7] Ben-Yehuda O., Kazi D.S., Bonafede M. (2016). Angina and associated healthcare costs following percutaneous coronary intervention: a real-world analysis from a multi-payer database. Cathet Cardio Intervent.

[8] Thomas M., Jones P.G., Arnold S.V., Spertus J.A. (2021). Interpretation of the Seattle angina questionnaire as an outcome measure in clinical trials and clinical care: a review. JAMA Cardiol.

[9] Tegn N., Abdelnoor M., Aaberge L. (2018). Health-related quality of life in older patients with acute coronary syndrome randomised to an invasive or conservative strategy. The after eighty randomised controlled trial. Age Ageing.

[10] Yang L.-X., Zhou Y.-J., Wang Z.-J., Li Y.-P., Chai M. (2014). Impact of invasive treatment strategy on health-related quality of life six months after non-ST-elevation acute coronary syndrome. J Geriatr Cardiol.

[11] Tao R., Zhang S., Huang X. (2019). Magnetocardiography-based ischemic heart disease detection and localization using machine learning methods. IEEE Trans. Biomed. Eng.

[12] Lim H.K., Chung N., Kim K. (2007). Can magnetocardiography detect patients with non-ST-segment elevation myocardial infarction?. Ann Med.

[13] Kyoon Lim H., Kim K., Lee Y.-H., Chung N. (2009). Detection of non-ST-elevation myocardial infarction using magnetocardiogram: new information from spatiotemporal electrical activation map. Ann Med.

[14] Hänninen H., Takala P., Korhonen P. (2002). Features of ST segment and T-wave in exercise-induced myocardial ischemia evaluated with multichannel magnetocardiography. Ann Med.

[15] Nomura M., Nakaya Y., Fujino K. (1989). Magnetocardiographic studies of ventricular repolarization in old inferior myocardial infarction. Eur Heart J.

[16] Fenici R., Brisinda D., Meloni A.M. (2005). Clinical application of magnetocardiography. Expert Rev Mol Diagn.

[17] Tolstrup K., Akhtari M., Brisinda D., Meloni A.M., Siegel R.J., Fenici R. (2025). Accurate diagnosis of ischemic heart disease without exposure to radiation using non-stress unshielded magnetocardiography. Am Heart J Plus.

[18] Bang W.-D., Kim K., Lee Y.-H. (2016). Repolarization heterogeneity of magnetocardiography predicts long-term prognosis in patients with acute myocardial infarction. Yonsei Med J.

[19] Korhonen P., Husa T., Tierala I. (2006). QRS duration in high-resolution methods and standard ECG in risk assessment after first and recurrent myocardial infarctions. Pacing Clin Electrophysiol.

[20] Korhonen P., Husa T., Tierala I. (2006). Increased Intra-QRS fragmentation in magnetocardiography as a predictor of arrhythmic events and mortality in patients with cardiac dysfunction after myocardial infarction. J Cardiovasc Electrophysiol.

[21] Zhang H., Ma Z., Mi H. (2024). Diagnostic value of magnetocardiography to detect abnormal myocardial perfusion: a pilot study. Rev Cardiovasc Med.

[22] Yang S., Feng L., Zhang M. (2024). Development and validation of a clinical diagnostic model for myocardial ischaemia in borderline coronary lesions based on optical pumped magnetometer magnetocardiography: a prospective observational cohort study. BMJ Open.

[23] Haberkorn W., Steinhoff U., Burghoff M., Kosch O., Morguet A., Koch H. (2006). Pseudo current density maps of electrophysiological heart, nerve or brain function and their physical basis. Biomagn Res Technol.

[24] Kimble L.P., Dunbar S.B., Weintraub W.S. (2002). The Seattle angina questionnaire: reliability and validity in women with chronic stable angina. Heart Dis.

[25] Park J., Hill P.M., Chung N., Hugenholtz P.G., Jung F. (2005). Magnetocardiography predicts coronary artery disease in patients with acute chest pain. Ann Noninvasive Electrocardiol.

[26] Pena M.E., Pearson C.L., Goulet M.P. (2020). A 90-second magnetocardiogram using a novel analysis system to assess for coronary artery stenosis in emergency department observation unit chest pain patients. Int J Cardiol Heart Vasc.

[27] Ashokprabhu N., Ziada K., Daher E. (2024). Evaluation of coronary microvascular dysfunction using magnetocardiography: a new application to an old technology. Am Heart J Plus.

[28] Mangiacapra F., Del Buono M.G., Abbate A. (2020). Role of endothelial dysfunction in determining angina after percutaneous coronary intervention: learning from pathophysiology to optimize treatment. Prog Cardiovasc Dis.

[29] Del Buono M.G., Montone R.A., Camilli M. (2021). Coronary Microvascular Dysfunction Across the Spectrum of Cardiovascular Diseases: JACC State-of-the-Art Review. J Am Coll Cardiol.

[30] Collison D., Copt S., Mizukami T. (2023). Angina after percutaneous coronary intervention: patient and procedural predictors. Circ Cardiovasc Interv.

