# Supplementary material for: Predictive Accuracy of Magnetocardiography for Diagnosing Myocardial Ischemia in NSTE-ACS Patients With Residual Post-PCI Angina
Source: JACC Adv. 2025 Aug 19;4(9):102073. doi: 10.1016/j.jacadv.2025.102073 (PMC12395062; doi:10.1016/j.jacadv.2025.102073)
Supplement: Supplemental_Material [file mmc4.pdf]

Supplemental Table 1. The definitions of MCG parameters

**Parameters**	**Definition**
RoART	The ratio of magnetic field amplitudes at R-peak and T-peak
RoART+	The ratio of magnetic field amplitude at R-peak and the positive amplitude at T-peak
RoART-	The ratio of magnetic field amplitude at R-peak and the negative amplitude at T-peak
RA	The magnetic field angle of the R-peak
RTA	The magnetic field angle between R-peak and T-peak
TA	The magnetic field angle of the T-peak
RoPNT	The ratio of positive to negative magnetic field amplitude at T-peak
TCAmax	The maximum current angle at T-peak
MAmin	The minimum magnetic field angle at intervals of a certain time τ within TT segment
MAmax	The maximum magnetic field angle at intervals of a certain time τ within TT segment
CAmax	The maximum current angle at intervals of a certain time τ within TT segment
CAmin	The minimum current angle at intervals of a certain time τ within TT segment

CMAsum	The sum of changes in magnetic field angle at intervals of a certain time τ within TT segment
CMAstd	The standard deviation of changes in magnetic field angle at intervals of a certain time τ within TT segment
CMAmax	The maximum value of changes in magnetic field angle at intervals of a certain time τ within TT segment
CMAmin	The minimum value of changes in magnetic field angle at intervals of a certain time τ within TT segment
CMPDsum	The sum of changes in magnetic pole distance at intervals of a certain time τ within TT segment
CMPDstd	The standard deviation of changes in magnetic pole distance at intervals of a certain time τ within TT segment
CMPDmax	The maximum value of changes in magnetic pole distance at intervals of a certain time τ within TT segment
CMPDmin	The minimum value of changes in magnetic pole distance at intervals of a certain time τ within TT segment
CCAsum	The sum of changes in current angle at intervals of a certain time τ within TT segment
CCAstd	The standard deviation of changes in current angle at intervals of a certain time τ within TT segment
CCAmax	The maximum value of changes in current angle at intervals of a certain time τ within TT segment

CCAmin	The minimum value of changes in current angle at intervals of a certain time τ within TT segment
CPPPPsum	The sum of changes in the position of the positive pole point at intervals of a certain time τ within TT segment
CPPPPstd	The standard deviation of changes in the position of the positive pole point at intervals of a certain time τwithin TT segment
CPPPPmax	The maximum value of changes in the position of the positive pole point at intervals of a certain time τ within TT segment
CPNPPsum	The sum of changes in the position of the negative pole point at intervals of a certain time τ within TT segment
CPNPPstd	The standard deviation of changes in the negative of the positive pole point at intervals of a certain time τ within TT segment
CPNPPmax	The maximum value of changes in the position of the negative pole point at intervals of a certain time τ within TT segment
CPCAsum	The sum of changes in the position of the current angle at intervals of a certain time τ within TT segment
CPCAstd	The standard deviation of changes in the position of the current angle at intervals of a certain time τ within TT segment
CPCAmax	The maximum value of changes in the position of the current angle at intervals of a certain time τ within TT segment
CPPAsum	The sum of changes in positive pole area at intervals of a certain time τ within TT segment

CPPAstd	The standard deviation of changes in positive pole area at intervals of a certain time τ within TT segment
CPPAmax	The maximum value of changes in positive pole area at intervals of a certain time τ within TT segment
CPPAmin	The minimum value of changes in positive pole area at intervals of a certain time τ within TT segment
CNPAsum	The sum of changes in negative pole area at intervals of a certain time τ within TT segment
CNPAstd	The standard deviation of changes in negative pole area at intervals of a certain time τ within TT segment
CNPAmax	The maximum value of changes in negative pole area at intervals of a certain time τ within TT segment
CNPAmin	The minimum value of changes in negative pole area at intervals of a certain time τ within TT segment
CPPPAsum	The sum of changes in positive pole point area at intervals of a certain time τ within TT segment
CPPPAstd	The standard deviation of changes in positive pole point area at intervals of a certain time τ within TT segment
CPPPAmax	The maximum value of changes in positive pole point area at intervals of a certain time τ within TT segment
CPPPAmin	The minimum value of changes in positive pole point area at intervals of a certain time τ within TT segment

CNPPAsum	The sum of changes in negative pole point area at intervals of a certain time τ within TT segment
CNPPAstd	The standard deviation of changes in negative pole point area at intervals of a certain time τ within TT segment
CNPPAmax	The maximum value of changes in negative pole point area at intervals of a certain time τ within TT segment
CNPPAmin	The minimum value of changes in negative pole point area at intervals of a certain time τ within TT segment
CPPATbp	The change in positive pole area between T-begin and T-peak
PPAmax	The maximum value of the positive pole area at intervals of a certain time τ within TT segment
PPAmin	The minimum value of the positive pole area at intervals of a certain time τ within TT segment
CNPATbp	The change in nagative pole area between T-begin and T-peak
NPAmax	The maximum value of the negative pole area at intervals of a certain time τ within TT segment
NPAmin	The minimum value of the negative pole area at intervals of a certain time τ within TT segment
CPPPATbp	The change in positive pole point area between T-begin and T-peak
PPPAmax	The maximum value of the positive pole point area at intervals of a certain time τ within TT segment

PPPAmin	The minimum value of the positive pole point area at intervals of a certain time τ within TT segment
CNPPATbp	The change in negative pole point area between T-begin and T-peak
NPPAmax	The maximum value of the negative pole point area at intervals of a certain time τ within TT segment
NPPAmin	The minimum value of the negative pole point area at intervals of a certain time τ within TT segment
CRAsum	The sum of changes in the ratio of positive to negative area at intervals of a certain time τ within TT segment
CRAstd	The standard deviation of changes in the ratio of positive to negative area at intervals of a certain time τ within TT segment
CRAmax	The maximum value of changes in the ratio of positive to negative area at intervals of a certain time τ within TT segment
CRAmin	The minimum value of changes in the ratio of positive to negative area at intervals of a certain time τ within TT segment

Ro = Ratio, R = R peak, T = T peak, M = Magnetic Field, C = Current, CP = Positive Pole, CN = Negative Pole, δ = Change value, A = Angle/ Magnitude/Area, D = Distance, P = Position, sum = Sum of all values, std = Standard deviation, max = Maximum, min = Minimum, bp = Baseline to post-P wave, TT = TT segment , Rp = R peak, Tp = T peak

Supplemental Table 2. Baseline, Follow-Up and Change in SAQ-7 scores

Variables	Total	Post-PCI angina	No post-PCI angina	P value
**Baseline SAQ-7**				
Angina Stability	62.33 ± 26.69	55.60 ± 24.26	66.27 ± 27.31	<0.001
Treatment Satisfaction	77.82 ± 17.25	70.34 ± 14.10	82.21 ± 17.44	<0.001
**Follow-up SAQ**				
Angina Stability	76.86 ± 24.83	64.55 ± 24.58	84.06 ± 22.02	<0.001
Treatment Satisfaction	82.37 ± 21.08	72.01 ± 23.76	88.43 ± 16.63	<0.001
**Change in SAQ Scores**				
Angina Stability	14.53 ± 27.19	8.96 ± 22.40	17.79 ± 29.20	0.003
Treatment Satisfaction	4.55 ± 19.31	1.68 ± 26.23	6.22 ± 13.53	0.030

Values are mean ± SD

SAQ = Seattle Angina Questionnaire

Supplemental Table 3. Delta OPM-MCG Parameters of Univariable Logistic Regression for Angina Prediction

Delta parameters	Post-PCI angina	No post-PCI angina	Odds Ratio	P value
RoART	1.42 (0.83, 2.00)	-0.94 (-1.38, -0.49)	1.305 (1.193-1.442)	<0.001
RoART+	0.42 (-3.93, 4.78)	-0.94 (-1.91, 0.04)	1.007 (0.992-1.031)	0.48
RoART-	4.81 (2.40, 7.22)	-5.31 (-8.97, -1.65)	1.080 (1.049-1.116)	<0.001
RA	-2.68 (-11.12, 5.77)	2.49 (-4.67, 9.65)	0.998 (0.994-1.002)	0.374
RTA	23.98 (10.50, 37.46)	-16.78 (-29.48, -4.09)	1.005 (1.003-1.008)	<0.001
TA	25.21 (12.94, 37.48)	-16.57 (-26.29, -6.86)	1.008 (1.005-1.011)	<0.001
RoPNT	0.97 (-0.34, 2.28)	-1.54 (-3.38, 0.31)	1.466 (1.209-1.833)	<0.001
TCAmax	14.75 (0.38, 29.12)	0.75 (-11.40, 12.89)	1.002 (0.999-1.004)	0.157
MAmin	16.63 (4.60, 28.66)	-10.48 (-20.00, -0.95)	1.005	0.001

			(1.002-1.008)	
MAmax	34.82 (22.38, 47.26)	-22.49 (-32.26, -12.72)	1.011 (1.007-1.015)	<0.001
CAmax	22.41 (8.19, 36.62)	-8.32 (-18.16, 1.51)	1.005 (1.002-1.008)	0.001
CAmin	-1.03 (-17.04, 14.98)	5.28 (-7.97, 18.54)	0.999 (0.997-1.002)	0.559
CMAsum	17.94 (10.17, 25.71)	-16.46 (-28.61, -4.31)	1.014 (1.008-1.021)	<0.001
CMAstd	4.44 (2.28, 6.59)	-3.81 (-6.06, -1.55)	1.061 (1.035-1.092)	<0.001
CMAmax	8.01 (2.29, 13.72)	-6.56 (-12.06, -1.06)	1.016 (1.007-1.028)	0.003
CMAmin	-6.66 (-11.32, -2.00)	7.58 (2.28, 12.88)	0.983 (0.972-0.992)	0.001
CMPDsum	20.95 (8.41, 33.49)	-11.48 (-21.68, -1.29)	1.006 (1.003-1.009)	<0.001
CMPDstd	4.14 (1.34, 6.94)	-2.21 (-4.42, 0.00)	1.024 (1.010-1.040)	0.001
CMPDmax	4.12 (-2.74, 10.98)	-4.51 (-11.14, 2.12)	1.004 (0.999-1.009)	0.103
CMPDmin	-11.08 (-18.68, -3.49)	3.05 (-0.71, 6.81)	0.988	0.001

			(0.981-0.995)	
CCAsum	29.77 (7.59, 51.94)	-14.35 (-32.21, 3.50)	1.003 (1.001-1.005)	0.006
CCAstd	6.92 (1.83, 12.01)	-3.86 (-7.49, -0.24)	1.014 (1.006-1.022)	0.001
CCAmax	15.86 (3.16, 28.57)	-7.22 (-15.88, 1.44)	1.005 (1.002-1.008)	0.004
CCAmin	-9.75 (-21.29, 1.79)	7.02 (-1.84, 15.88)	0.996 (0.993-0.999)	0.029
CPPPPsum	40.00 (18.95, 61.04)	-15.66 (-31.35, 0.04)	1.004 (1.002-1.006)	<0.001
CPPPPstd	10.96 (4.86, 17.06)	-4.16 (-8.29, -0.02)	1.014 (1.007-1.022)	<0.001
CPPPPmax	35.63 (16.15, 55.10)	-13.85 (-27.03, -0.68)	1.005 (1.002-1.007)	<0.001
CPNPPsum	43.82 (20.56, 67.08)	-39.31 (-62.98, -15.65)	1.004 (1.002-1.006)	<0.001
CPNPPstd	9.29 (3.36, 15.22)	-9.53 (-14.75, -4.31)	1.016 (1.008-1.024)	<0.001
CPNPPmax	30.88 (12.05, 49.70)	-30.47 (-45.72, -15.22)	1.005 (1.003-1.008)	<0.001
CPCAsum	31.45 (-4.51, 67.42)	-21.31 (-42.62, -0.01)	1.002	0.012

			(1.000-1.003)	
CPCAstd	4.94 (-2.34, 12.23)	-3.26 (-7.99, 1.47)	1.005 (1.000-1.011)	0.057
CPCAmax	-34.32 (-131.32, 62.67)	-9.31 (-23.86, 5.25)	1.000 (0.999-1.000)	0.543
CPPAsum	2465.08 (-1300.04, 6230.20)	-1244.97 (-4458.84, 1968.90)	1.000 (1.000-1.000)	0.158
CPPAstd	204.22 (-207.74, 616.18)	-187.18 (-506.23, 131.88)	1.000 (1.000-1.000)	0.149
CPPAmax	624.41 (-712.06, 1960.89)	-329.47 (-1211.38, 552.43)	1.000 (1.000-1.000)	0.229
CPPAmin	-714.87 (-1594.54, 164.79)	423.96 (-317.72, 1165.64)	1.000 (1.000-1.000)	0.065
CNPAsum	6561.41 (2964.91, 10157.92)	-3130.30 (-5725.27, -535.32)	1.000 (1.000-1.000)	<0.001
CNPAstd	606.29 (263.88, 948.70)	-163.96 (-454.04, 126.13)	1.000 (1.000-1.000)	0.003
CNPAmax	1420.37 (362.73, 2478.01)	-196.07 (-1138.13, 745.99)	1.000 (1.000-1.000)	0.039
CNPAmin	-1338.42 (-2110.60, -566.24)	614.71 (-38.65, 1268.07)	1.000 (1.000-1.000)	0.001
CPPPAsu	2672.85 (285.97,	-828.26 (-2165.53,	1.000	0.01

m	5059.73)	509.02)	(1.000-1.000)	
CPPPAstd	262.99 (-21.13, 547.10)	-99.08 (-256.05, 57.90)	1.000 (1.000-1.000)	0.024
CPPPAmax	619.49 (-145.16, 1384.15)	-265.77 (-729.99, 198.45)	1.000 (1.000-1.000)	0.046
CPPPAmin	-977.21 (-1691.46, -262.96)	167.02 (-196.19, 530.24)	1.000 (1.000-1.000)	0.005
CNPPAsum	2372.99 (866.72, 3879.27)	-1039.30 (-1936.10, -142.50)	1.000 (1.000-1.000)	<0.001
CNPPAstd	275.76 (108.03, 443.50)	-122.86 (-231.31, -14.42)	1.001 (1.000-1.001)	<0.001
CNPPAmax	695.75 (215.21, 1176.30)	-227.54 (-559.87, 104.80)	1.000 (1.000-1.000)	0.003
CNPPAmin	-544.96 (-978.81, -111.11)	288.23 (45.97, 530.49)	1.000 (1.000-1.000)	0.001
CPPATbp	391.90 (-4661.37, 5445.18)	-442.61 (-4194.24, 3309.02)	1.000 (1.000-1.000)	0.793
PPAmax	-4684.90 (-11352.73, 1982.94)	9006.57 (3921.31, 14091.84)	1.000 (1.000-1.000)	0.002
PPAmin	-6202.93 (-12351.36, -54.49)	10136.37 (5205.18, 15067.56)	1.000 (1.000-1.000)	<0.001
CNPATbp	-340.55 (-4791.77,	377.19 (-2993.80,	1.000	0.8

	4110.67)	3748.17)	(1.000-1.000)	
NPAmax	12120.89 (6996.96, 17244.82)	-9710.59 (-13976.52, -5444.66)	1.000 (1.000-1.000)	<0.001
NPAmin	7175.99 (2659.84, 11692.15)	-7064.25 (-10579.73, -3548.77)	1.000 (1.000-1.000)	<0.001
CPPPATbp	-217.08 (-1766.04, 1331.89)	-41.04 (-1024.41, 942.32)	1.000 (1.000-1.000)	0.843
PPPAmax	390.90 (-1564.72, 2346.52)	812.92 (-632.36, 2258.20)	1.000 (1.000-1.000)	0.731
PPPAmin	-691.88 (-1604.99, 221.23)	1421.60 (499.90, 2343.31)	1.000 (1.000-1.000)	0.004
CNPPATbp	128.25 (-756.04, 1012.55)	-58.08 (-765.11, 648.96)	1.000 (1.000-1.000)	0.749
NPPAmax	2160.64 (759.13, 3562.16)	-515.06 (-1448.50, 418.39)	1.000 (1.000-1.000)	0.002
NPPAmin	535.84 (-240.62, 1312.30)	242.41 (-222.62, 707.44)	1.000 (1.000-1.000)	0.499
CRAsum	-0.42 (-0.94, 0.10)	0.23 (-0.01, 0.47)	0.862 (0.750-0.967)	0.024
CRAstd	-0.04 (-0.15, 0.06)	0.03 (0.00, 0.05)	0.536 (0.129-1.093)	0.261
CRAmax	0.05 (-0.04, 0.13)	-0.01 (-0.05, 0.02)	1.554	0.154

			(0.864-2.975)	
CRAmin	0.17 (-0.17, 0.51)	-0.09 (-0.18, 0.00)	1.359<br>(1.008-2.293)	0.177

Ro = Ratio, R = R peak, T = T peak, M = Magnetic Field, C = Current, CP = Positive Pole, CN = Negative Pole, δ = Change value, A = Angle/ Magnitude/Area, D = Distance, P = Position, sum = Sum of all values, std = Standard deviation, max = Maximum, min = Minimum, bp = Baseline to post-P wave, TT = TT segment , Rp = R peak, Tp = T peak

Supplemental Table 4 . Bootstrap Selection Frequencies and Coefficient Estimates of ΔParameters Predictors for Angina

**Predictor**	**Selected /1 000**	**%**	**Median β**	**95 % CI β**
ΔRoART	987	98.7	0.127	0.021-0.350
ΔNPAmax	885	88.5	0.000	0.000-0.000
ΔCPPPPmax	849	84.9	0.001	0.000-0.012
ΔMAmax	760	76	0.003	0.000-0.008
ΔCAmax	713	71.3	0.002	0.000-0.019

Supplemental Table 5. Pre- and post-PCI OPM-MCG Parameters of Univariable Logistic Regression for Angina Prediction

Parameters	Post-PCI angina	No post-PCI angina	Odds Ratio	P value
**Pre-PCI parameters**				
RoART	3.72 (2.82, 5.56)	3.56 (2.58, 5.11)	0.91 (0.83 - 0.99)	0.04
RoART-	8.00 (5.07, 13.04)	6.17 (3.90, 9.70)	0.97 (0.94 - 0.99)	0.02
TA	-43.38 (-68.89, 16.16)	-62.80 (-71.27, -33.50)	1.00 (0.99 - 1.00)	0.01
RoPNT	0.89 (0.59, 1.54)	0.69 (0.51, 0.98)	0.77 (0.61 - 0.93)	0.01
MAmin	-37.30 (-66.37, 24.14)	-62.56 (-70.99, -16.62)	0.99 (0.99 - 1.00)	<0.001
CAmax	54.29 (35.71, 104.04)	41.05 (25.82, 60.64)	1.00 (0.99 - 1.00)	0.03
CMAsum	13.23 (6.02, 36.48)	6.10 (2.83, 15.02)	0.99 (0.98 - 1.00)	0.03
CMAstd	0.98 (0.35, 7.34)	0.41 (0.20, 1.22)	0.97 (0.93 - 0.99)	0.03
CMAmax	1.39 (0.53, 5.71)	0.83 (0.41, 2.03)	0.98 (0.97 -	0.04

			1.00)	
CCAsum	19.40 (8.08, 54.64)	14.32 (8.30, 25.61)	1.00 (0.99 - 1.00)	0.02
CCAstd	1.47 (0.82, 10.89)	1.25 (0.81, 2.68)	0.98 (0.97 - 1.00)	0.02
CCAmax	2.87 (1.20, 8.71)	3.45 (1.44, 5.38)	0.99 (0.99 - 1.00)	0.04
CPNPPsum	48.30 (26.03, 117.02)	27.57 (14.06, 53.98)	1.00 (1.00 - 1.00)	0.01
CPNPPstd	2.98 (1.26, 17.50)	1.44 (0.91, 3.54)	0.99 (0.98 - 1.00)	0.02
CPNPPmax	11.40 (5.00, 59.20)	5.52 (3.16, 13.12)	1.00 (0.99 - 1.00)	0.01
CPPAmin	-1693.00 (-3539.00, -792.00)	-1443.50 (-2792.25, -382.75)	1.00 (1.00 - 1.00)	0.04
CNPAsum	14961.00 (8842.00, 24131.00)	9663.00 (5987.75, 16764.50)	1.00 (1.00 - 1.00)	<0.001
CNPAstd	824.45 (495.38, 1571.22)	528.06 (312.60, 1057.64)	1.00 (1.00 - 1.00)	0.02
CNPAmax	2303.00 (1010.00, 4312.00)	1432.00 (505.00, 3041.75)	1.00 (1.00 - 1.00)	0.02
CNPPAsum	2534.00 (1049.00,	1408.50 (684.75,	1.00 (1.00 -	0.01

	6214.00)	3403.75)	1.00)	
CNPPAstd	198.65 (76.14, 611.34)	99.93 (47.37, 252.18)	1.00 (1.00 - 1.00)	0.02
CNPPAmax	492.00 (159.00, 1480.00)	237.00 (88.75, 663.50)	1.00 (1.00 - 1.00)	0.03
PPAmin	83009.00 (61150.00, 103206.00)	89901.00 (68750.50, 107462.25)	1.00 (1.00 - 1.00)	0.02
NPAmax	62735.00 (49807.00, 82426.00)	53008.50 (40694.00, 68707.50)	1.00 (1.00 - 1.00)	<0.001
NPAmin	46541.00 (35846.00, 60600.00)	41746.50 (29064.00, 55954.00)	1.00 (1.00 - 1.00)	<0.001
CRAsum	0.64 (0.38, 1.26)	0.62 (0.33, 1.52)	1.21 (1.04 - 1.46)	0.03
**Post- PCI parameters**				
RoART	3.23 (2.55, 4.63)	4.38 (3.36, 6.06)	1.31 (1.19 - 1.47)	<0.001
RoART+	7.69 (5.00, 11.11)	9.52 (6.88, 13.48)	1.07 (1.04 - 1.11)	<0.001
RoART-	5.88 (4.30, 9.70)	9.25 (5.92, 15.48)	1.08 (1.04 - 1.12)	<0.001
RA	-62.77 (-71.89, -43.03)	-65.00 (-78.16, -41.10)	0.99 (0.99 - 1.00)	0.05

RTA	-0.47 (-22.04, 21.78)	28.20 (-9.62, 87.02)	1.01 (1.00 - 1.01)	<0.001
TA	-62.85 (-71.45, -42.63)	-26.68 (-66.98, 24.19)	1.01 (1.00 - 1.01)	<0.001
MAmax	-68.48 (-76.10, -52.56)	-53.77 (-77.60, 6.00)	1.01 (1.00 - 1.01)	<0.001
MAmin	-61.78 (-70.82, -37.16)	-1.03 (-62.07, 29.58)	1.01 (1.01 - 1.01)	<0.001
CAmax	45.00 (31.50, 72.65)	75.96 (41.88, 114.74)	1.01 (1.00 - 1.01)	0.01
CMAsum	7.56 (3.63, 18.81)	19.64 (7.21, 47.03)	1.01 (1.00 - 1.02)	<0.001
CMAstd	0.43 (0.24, 1.09)	1.62 (0.42, 8.64)	1.05 (1.03 - 1.09)	<0.001
CMAmax	1.21 (0.48, 2.85)	1.67 (0.37, 5.49)	1.01 (1.00 - 1.02)	0.04
CMAmin	-0.17 (-1.02, 0.18)	-1.02 (-7.61, -0.05)	0.98 (0.96 - 0.99)	<0.001
CMPDsum	22.25 (12.60, 42.74)	42.86 (23.29, 87.75)	1.01 (1.00 - 1.01)	<0.001
CMPDstd	1.61 (0.95, 3.57)	4.17 (1.64, 16.17)	1.03 (1.01 - 1.05)	<0.001

CMPDmin	-2.79 (-7.14, -1.20)	-4.68 (-17.04, -1.38)	0.98 (0.97 - 0.99)	<0.001
CCAstd	1.39 (0.80, 3.15)	1.84 (0.78, 9.65)	1.01 (1.00 - 1.02)	0.02
CCAmax	2.53 (1.24, 6.86)	2.82 (1.20, 9.04)	1.00 (1.00 - 1.01)	0.05
CPPPPsum	24.15 (14.89, 46.54)	43.13 (17.61, 130.82)	1.00 (1.00 - 1.01)	<0.001
CPPPPstd	1.21 (0.86, 2.41)	2.26 (1.12, 16.88)	1.02 (1.01 - 1.03)	<0.001
CPPPPmax	5.00 (3.16, 10.30)	9.14 (4.12, 57.61)	1.01 (1.00 - 1.01)	<0.001
CPNPPsum	33.77 (17.37, 69.00)	58.34 (25.21, 135.09)	1.00 (1.00 - 1.00)	0.01
CPNPPstd	1.75 (0.91, 3.98)	2.69 (1.36, 19.43)	1.01 (1.00 - 1.02)	<0.001
CPNPPmax	7.07 (3.16, 16.55)	11.18 (5.10, 65.96)	1.00 (1.00 - 1.01)	<0.001
CNPAsum	11244.00 (6915.00, 19903.00)	13174.50 (8728.25, 24487.00)	1.00 (1.00 - 1.00)	0.03
CNPAmin	-200.00 (-1719.00, 456.00)	-1256.00 (-3264.50, -214.50)	1.00 (1.00 - 1.00)	<0.001

CPPPAsum	2259.00 (1106.00, 5482.00)	3167.00 (1263.00, 9560.25)	1.00 (1.00 - 1.00)	0.01
CPPPAstd	156.00 (78.14, 374.04)	244.87 (87.42, 747.23)	1.00 (1.00 - 1.00)	0.02
CPPPAmax	279.00 (93.00, 887.00)	413.50 (137.00, 2003.50)	1.00 (1.00 - 1.00)	0.01
CPPPAmin	-251.00 (-640.00, -64.00)	-396.00 (-1460.75, -101.50)	1.00 (1.00 - 1.00)	0.01
CNPPAsum	1427.00 (723.00, 3573.00)	2487.00 (1074.50, 6316.50)	1.00 (1.00 - 1.00)	0.02
CNPPAstd	106.85 (44.59, 283.59)	188.05 (75.04, 680.46)	1.00 (1.00 - 1.00)	0.01
CNPPAmax	266.00 (116.00, 743.00)	411.00 (136.25, 1641.50)	1.00 (1.00 - 1.00)	0.04
CNPPAmin	-80.00 (-367.00, -5.00)	-344.00 (-1174.50, -85.00)	1.00 (1.00 - 1.00)	<0.001
PPAmax	110228.00 (86197.00, 124344.00)	101895.50 (75384.00, 120802.75)	1.00 (1.00 - 1.00)	0.03
PPAmin	96867.00 (67750.00, 114826.00)	81890.00 (58425.75, 107534.25)	1.00 (1.00 - 1.00)	0.01
NPAmax	53718.00 (43156.00,	65390.00 (50379.25,	1.00 (1.00 -	<0.001

	70660.00)	85457.50)	1.00)	
NPAmin	42177.00 (31970.00, 50567.00)	48168.50 (36529.75, 63880.00)	1.00 (1.00 - 1.00)	<0.001
PPPAmin	6444.00 (4130.00, 8906.00)	5042.00 (3128.50, 7652.75)	1.00 (1.00 - 1.00)	0.01
NPPAmax	4534.00 (3045.00, 7612.00)	5277.00 (3391.50, 9947.50)	1.00 (1.00 - 1.00)	0.04
CRAmax	0.01 (-0.03, 0.08)	0.06 (0.01, 0.17)	2.58 (1.13 - 6.78)	0.03

Ro = Ratio, R = R peak, T = T peak, M = Magnetic Field, C = Current, CP = Positive Pole, CN = Negative Pole, δ = Change value, A = Angle/ Magnitude/Area, D = Distance, P = Position, sum = Sum of all values, std = Standard deviation, max = Maximum, min = Minimum, bp = Baseline to post-P wave, TT = TT segment , Rp = R peak, Tp = T peak

Supplemental Table 6. Restricted Cubic-Spline Analysis of Δ-MCG Parameters and Post-PCI Angina

Parameters	Wald χ^2 (total effect)	P (total)	Wald χ^2 (non-linear component)	P (non-linear)
ΔRoART	13.60	0.004	0.33	0.85
ΔNPAmax	15.96	0.001	0.28	0.869
ΔMAmax	3.48	0.324	2.81	0.245
ΔCPPPPmax	8.02	0.046	3.89	0.143
ΔCAmax	8.07	0.045	5.94	0.051

Ro = Ratio, R = R peak, T = T peak, M = Magnetic Field, C = Current, CP = Positive Pole, CN = Negative Pole, δ = Change value, A = Angle/ Magnitude/Area, D = Distance, P = Position, sum = Sum of all values, std = Standard deviation, max = Maximum, min = Minimum, bp = Baseline to post-P wave, TT = TT segment , Rp = R peak, Tp = T peak

Supplemental Table 7. Calibration Intercept and Slope by Subgroup

Subgroup	n	Intercept	Slope
Overall	363	<0.001	1.031
Sex: Female	84	0.298	1.648
Sex: Male	279	-0.085	0.904
BMI: <26	150	-0.292	1.273
BMI: ≥26	250	0.261	0.857

BMI = Body Mass Index

Supplemental Figure 1. Real-time raw MCG signals and the sensors' power spectral density

A.

B.

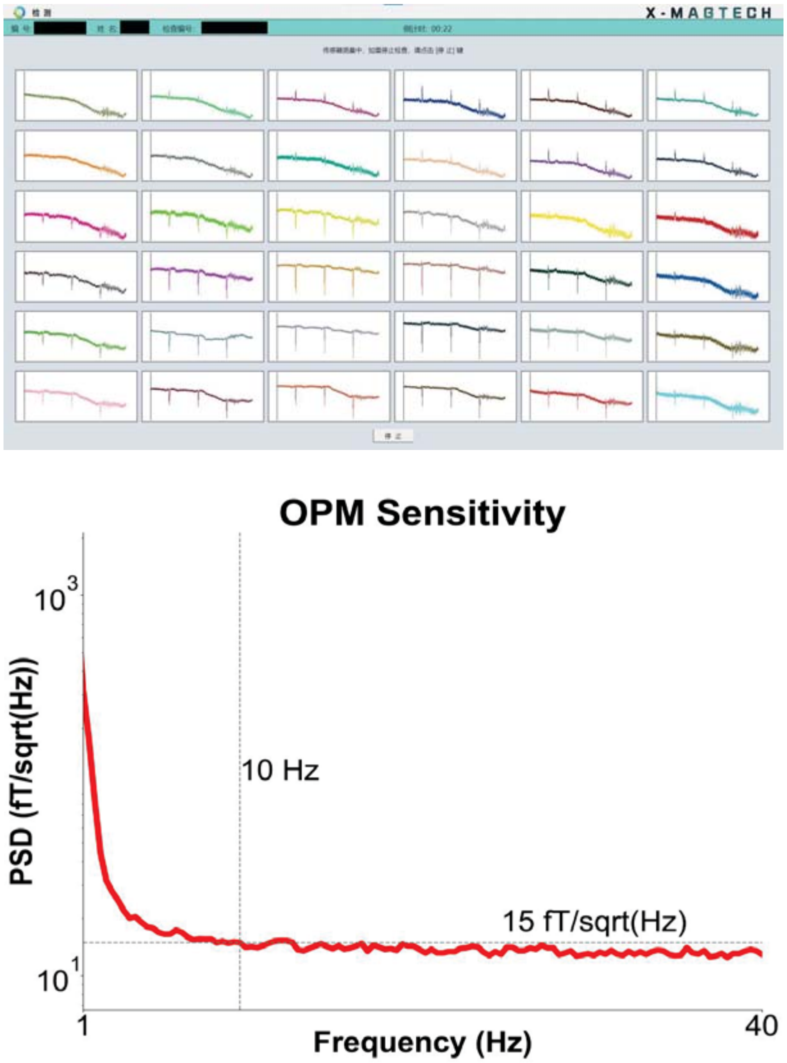

(A) Real-time Raw MCG Signals

(B) The Sensors' Power Spectral Density

OPM = Optical Pumped Magnetometer, MCG = Magnetocardiography

Supplemental Figure 2. Calibration curve and LASSO regularization path for the post-PCI angina prediction model

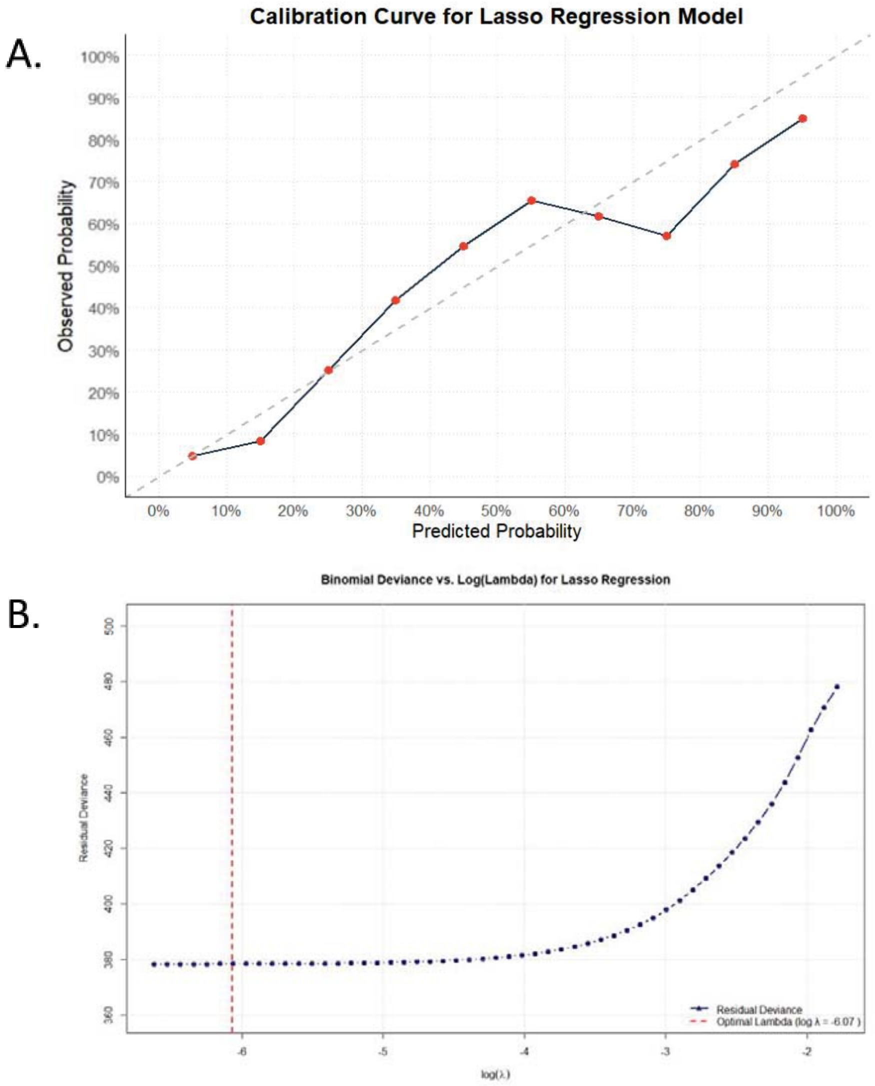

(A) Calibration Curve for LASSO Regression Model

(B) Binomial Deviance vs. Log(Lambda) for LASSO Regression

ROC = Receiver Operating Characteristic; LASSO = Least Absolute Shrinkage and Selection Operator

Supplemental Figure 3. Diagnostic Performance of Pre- and Post-PCI Parameters for Angina and MACCE

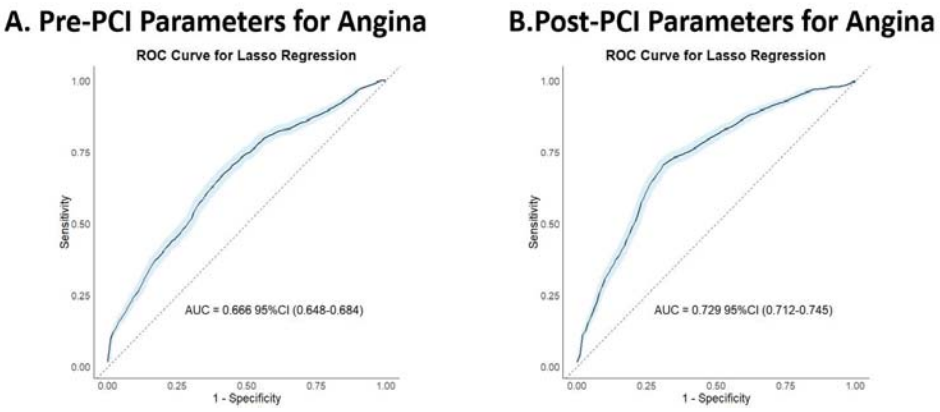

(A) ROC Curves of pre-PCI Parameters in Angina Prediction.

(B) ROC Curves of post-PCI Parameters in Angina Prediction.

ROC = Receiver Operating Characteristic; LASSO = Least Absolute Shrinkage and Selection Operator;

Supplemental Figure 4. ROC Curve for Elastic-Net Model Predicting Post-PCI Angina

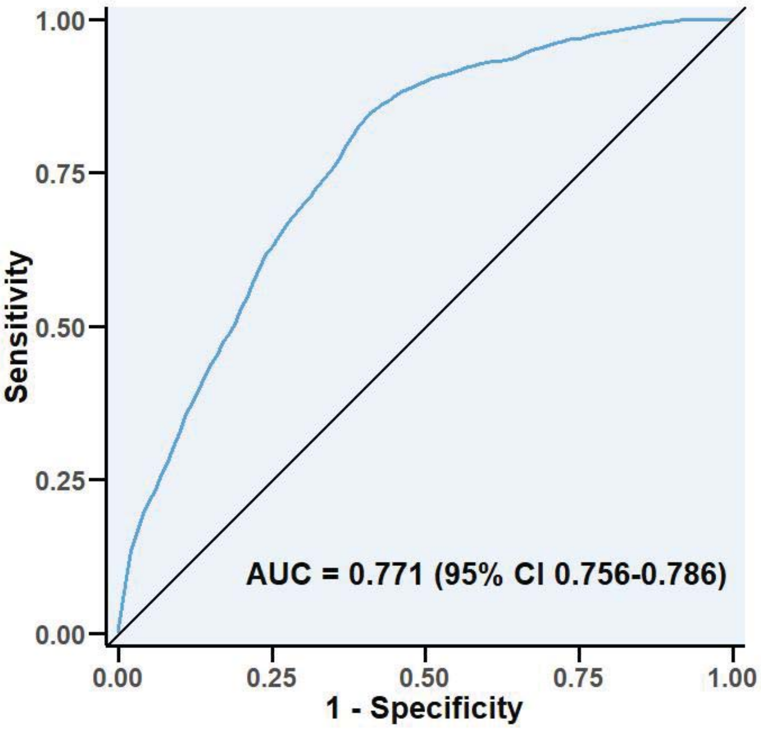

ROC = Receiver Operating Characteristic; AUC = Area Under Curve

Supplemental Figure 5. Restricted cubic-spline relationships between each Δparameter and the predicted probability of post-PCI angina.

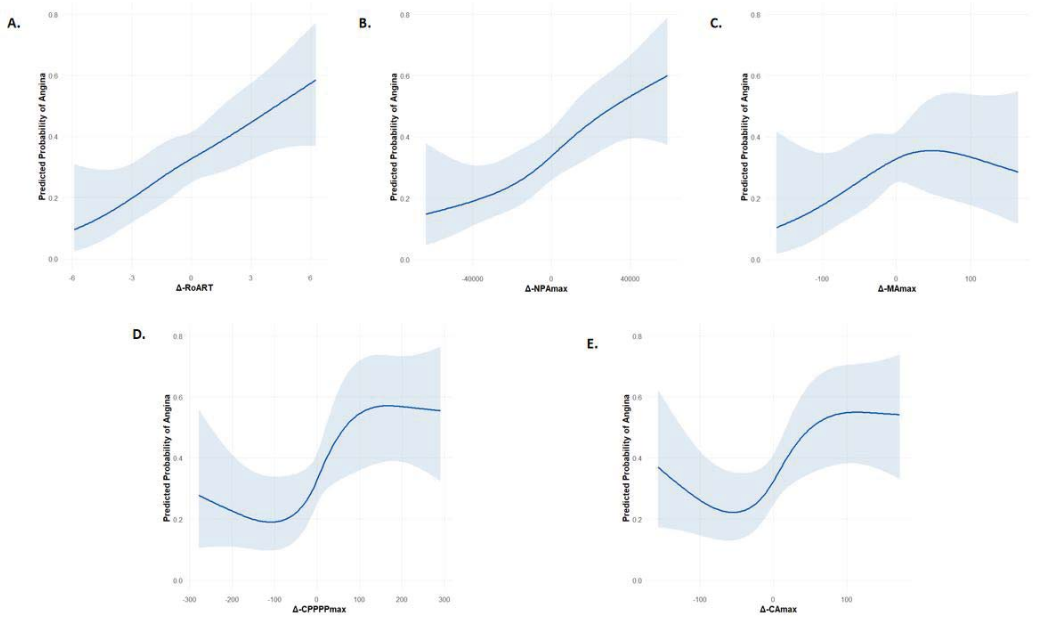

(A) Spline Curve of ΔRoART and Predicted Risk.

(B) Spline Curve of ΔNPAmax and Predicted Risk.

(C) Spline Curve of ΔMAmax and Predicted Risk.

(D) Spline Curve of ΔCPPPPmax and Predicted Risk.

(E) Spline Curve of ΔCAmax and Predicted Risk.

Shaded areas represent 95 % confidence intervals.

PCI = Percutaneous Coronary Intervention

Supplemental Figure 6. Coefficient and Permutation Importance of ΔParameters Predictors

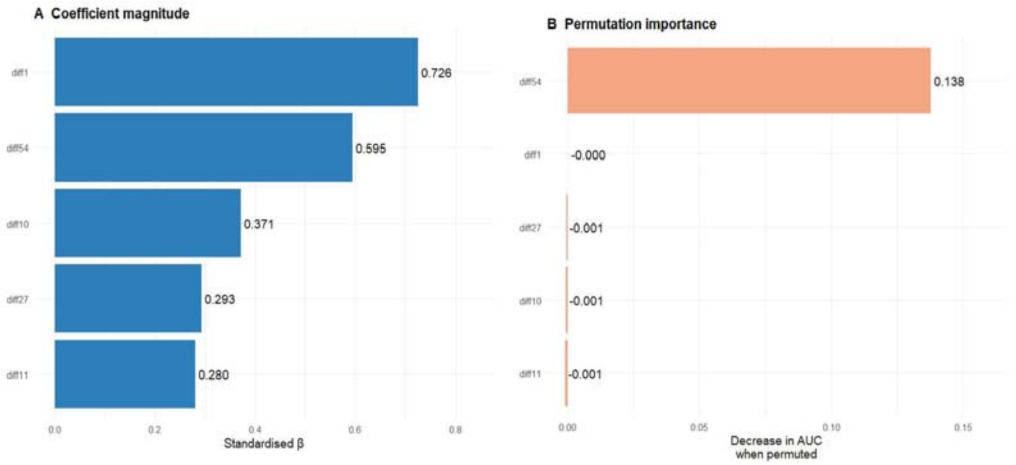

AUC = Area Under Curve
